# Sagittal-Plane Knee Flexion Moment Estimation Using a Lightweight Deep Learning Framework Based on Sequential Surface EMG Feature Frames

**DOI:** 10.3390/s26082500

**Published:** 2026-04-18

**Authors:** Yuanzhi Zhuo, Adrian Pranata, Chi-Tsun Cheng, Toh Yen Pang

**Affiliations:** 1 Biomedical Engineering Department, School of Engineering, STEM College, RMIT University, Melbourne, VIC 3000, Australia; yuanzhi.zhuo@rmit.edu.au; 2 School of Health and Biomedical Science, RMIT University, Bundoora, VIC 3082, Australia; adrian.pranata@rmit.edu.au; 3 Mechanical, Manufacturing and Mechatronic Engineering Department, School of Engineering, STEM College, RMIT University, Melbourne, VIC 3000, Australia; ben.cheng@rmit.edu.au

**Keywords:** knee moment, joint moment estimation, surface electromyography, continuous motion estimation, feature engineering, deep learning, gait analysis, microcontroller, wearable sensor

## Abstract

Knee joint moment is an important biomechanical parameter for sports assessment, rehabilitation monitoring, and human–machine interaction. However, direct measurement is often restricted to laboratory-based settings. Surface electromyography (sEMG) offers a non-invasive alternative for indirect joint moment estimation, but many existing deep learning models remain too computationally demanding for potential wearable edge deployment. To address this gap, this study proposes Topo2DCNN-LSTM, a lightweight two-dimensional (2D) convolutional neural network model, designed for sagittal-plane knee flexion moment estimation. The model used a feature-based sequential representation, transforming raw sEMG signals into compact Root Mean Square (RMS) feature frames. The input was processed by a lightweight 2D convolutional neural network (CNN) encoder and paired with long short-term memory (LSTM) units. The model was trained on a public walking dataset of healthy subjects with synchronized sEMG and joint kinetics at two treadmill speeds. When compared with selected deep learning baselines, the quantized model achieved a mean RMS Error of 0.088 ± 0.020 Nm/kg at 1.2 m/s and 0.114 ± 0.034 Nm/kg at 1.8 m/s. On a SparkFun Thing Plus–SAMD51, it achieved an average inference latency of 28 ms using 71,316 bytes of random-access memory (RAM) and 257,172 bytes of flash. These results support its use as a proof of concept for personalized unilateral knee moment estimation with isolated on-device inference feasibility under resource-constrained and limited walking conditions.

## 1. Introduction

Knee moment is a key measure in locomotion analysis in rehabilitation, sports performance, and human-machine interfaces. In rehabilitation, knee joint kinetics provides essential information for monitoring patient recovery and evaluating surgical outcomes [1]. In sports biomechanics, knee moment analysis aids in injury prevention and performance optimization [2,3]. Furthermore, knee moment estimation is increasingly important for the design of exoskeletons or prostheses [4]. Traditionally, the accurate measurement of knee joint moment relies on inverse dynamics, which is considered as the gold-standard methodology in current research [5]. However, inverse dynamics requires specialized equipment and laboratory settings, such as motion capture systems and force plates, making it unsuitable for continuous real-world monitoring.

Surface electromyography (sEMG) is a promising wearable modality for estimating joint mechanics because it provides a direct measure of neuromuscular activation that is related to joint mechanical output [6]. However, translating sEMG into reliable kinetic estimates typically requires careful signal processing, model calibration, and system integration. These steps can limit routine use outside specialist settings. Recent advances in deep learning have nevertheless motivated sEMG-based approaches for estimating joint kinematics and kinetics by learning nonlinear, time-dependent relationships between multi-channel muscle activation patterns and biomechanical outputs [5,7,8,9]. These developments are particularly relevant to ambulatory motion analysis.

A range of deep learning architectures has been investigated for sEMG-based biomechanics estimation. Recurrent neural networks, particularly long short-term memory (LSTM) networks, are widely used for sequence modelling because they capture temporal dependencies across multiple time scales [10]. Truong et al. [5] used an LSTM with time-domain sEMG features to estimate lower-limb joint angles and moments during functional tasks and reported an average coefficient of determination (*R*^2^) of 0.949. Song et al. [11] applied an LSTM to root mean square (RMS) features for online prediction of lower-limb joint angles and reported a knee-angle Root Mean Square Error (RMSE) of 2.81 ± 0.92 degrees. Bidirectional LSTM (BiLSTM) models have also been explored because they can use both past and future information within a windowed segment. For example, Zangene et al. [12] proposed an attention-enhanced BiLSTM for sEMG-based knee-angle estimation during running and reported a correlation coefficient of 0.984. Ma et al. [13] employed a BiLSTM to estimate continuous upper-limb joint movements under weakly synchronized conditions and reported a higher mean correlation coefficient of 0.8374 ± 0.0455 than LSTM and CNN models. In addition, a gated recurrent unit (GRU) provides a lighter alternative to LSTM, with fewer parameters and faster training while preserving similar sequence-modelling capability [14]. Ilyas et al. [15], for example, reported an average *R*^2^ of 0.921 ± 0.067 for sEMG-based finger-angle estimation using a GRU-based model. Convolutional neural networks (CNNs) have also been used to extract local inter-channel features from multi-channel sEMG [16], and hybrid CNN-LSTM models have been investigated for knee-joint movement estimation [17,18]. Temporal convolutional networks (TCNs), which use stacked one-dimensional dilated convolutions, have also shown potential for efficient long-range temporal modelling in finger kinematics estimation [19]. Together, these studies support the feasibility of deep learning for sEMG-based knee biomechanics estimation.

However, model performance depends not only on architecture, but also on how multi-channel sEMG is represented at the input. Recent work has represented multi-channel sEMG signals as image-like inputs to enable 2D CNNs to learn structured inter-channel relationships for classification and regression tasks [20,21,22,23,24,25]. In continuous motion estimation, this idea can be extended to a frame-based representation, in which the multi-channel sEMG pattern at each time point is arranged into a 2D frame and consecutive frames are stacked over time within a sliding window. Prior studies have used such sequential frame representation based on the anatomical distribution of target muscles or the sensor grid layout for hand gesture recognition and wrist angle prediction [21,24,25]. Nevertheless, these methods typically use raw signal inputs together with three-dimensional CNNs (3D CNNs) or multi-stream 2D CNN pipelines, which increase input size and computational burden. These factors can become major barriers to wearable or resource-constrained deployment.

Despite the growing progress in sEMG-based joint biomechanics estimation, translation to wearable and real-world use remains limited. Most published models are developed and evaluated on GPUs or CPUs, with relatively little attention to the memory and latency constraints of embedded systems. Although embedded EMG research is increasing, it has focused mainly on signal processing and classification rather than continuous regression of joint kinetics, leaving a deployment gap for EMG-driven joint moment estimation on resource-constrained devices [26,27]. For wearable monitoring, transmitting raw signals for remote processing can be inefficient and may introduce communication delay, whereas local inference can reduce communication overhead and support lower-latency feedback [28]. This, however, shifts the burden to model and system design under strict hardware limits.

Edge inference executes pre-trained models directly on portable, resource-limited devices instead of relying on cloud computation [29]. Although cloud processing can support offline analysis, latency and connectivity issues limit real-time use. Tiny machine learning (TinyML) addresses this by enabling deep learning inference on embedded or edge devices close to where data are generated [30]. The main challenge is balancing estimation accuracy with strict memory and latency constraints, which must be considered during model design. Consequently, accurate knee moment estimation during walking remains difficult for wearable biomechanics monitoring systems [8]. In particular, it remains unclear whether lightweight architectures can achieve a favorable trade-off between estimation accuracy and computational efficiency, and how well they are suited to model compression techniques such as post-training quantization [31].

To address this gap, this study proposes Topo2DCNN-LSTM, a lightweight 2DCNN-LSTM framework for knee flexion moment estimation from wearable sEMG sensors using a feature-based sequential frame representation. The present work builds on a previously reported frame-based sEMG representation approach by adapting it from raw sEMG samples to compact RMS feature frames. It further combines a compact 2D CNN encoder with an LSTM to support resource-constrained deployment. The aim is to determine whether Topo2DCNN-LSTM could improve personalized sagittal-plane knee flexion moment estimation compared with selected deep learning baselines while remaining suitable for isolated on-device inference. The main contributions are:

- We adapted a prior frame-based sEMG representation approach [21] into a lightweight feature-based deep learning framework by embedding sparse multi-channel RMS features, rather than raw sEMG samples, into a fixed 2D frame at each time step. The resulting frame sequence was then processed using a 2D CNN encoder and an LSTM to model spatiotemporal information.
- We evaluated the proposed model against conventional baselines without the frame-based representation, including LSTM, BiLSTM, TCN, 2DCNN-GRU, and 2DCNN-LSTM under similar model complexity based on a public dataset across 11 healthy subjects at two walking speeds.
- We applied a TinyML technique and assessed the feasibility of deploying the proposed model to a microcontroller unit (MCU), the SparkFun Thing Plus-SAMD51 (32-bit ARM Cortex-M4F, up to 120 MHz, 1 MB flash, 256 kB internal SRAM), including model inference time, flash memory and RAM usage.

## 2. Related Work

Recent studies have shown that transforming multi-channel sEMG into image-like inputs can improve feature learning in CNN-based models. However, prior work in this area generally follows two different directions: feature-image representations, which organize hand-crafted features into a 2D image or matrix per sliding window, and frame-based representations, which map instantaneous multi-channel sEMG samples into grid-like frames per sliding window according to electrode placement or anatomical distributions of the target muscles.

The first direction is feature-image representation. Hu et al. [22] proposed a feature-vector-based sEMG image representation that helped deep models capture implicit inter-channel correlations for sparse multi-channel gesture classification. Wang et al. [32] used spectrum-domain features from sEMG signals for a 2DCNN-based model and outperformed the benchmark for 7% overall accuracy in hand gesture recognition. Similar formulations have also been applied to continuous regression. Bao et al. [23] transformed sEMG into time- and spectrum-domain 2D images for continuous wrist angle estimation, where the feature image combined mean absolute value, RMS, variance, and fourth-order autoregressive coefficients and was modeled using a four-layer 2D CNN encoder. Liu et al. [20] compared a feature-based 6 × 4 image (six muscles × four features: MAV, RMS, waveform length, and energy percentage) with a raw 6 × 150 signal image (six muscles × window length) for knee angle estimation during gait, reporting a 44% RMSE reduction and a 5.2% increase in *R*^2^ with the feature image. Collectively, these studies suggest that feature-based 2D representations can be more compact and informative than direct raw-signal 2D images. However, in most cases, the input remains a single 2D matrix per window, rather than an explicit sequence of frames over time.

The second direction is frame-based representation. Specifically, multi-channel sEMG samples at each time instant are mapped into 2D frames according to channel layout, and frame sequences are then processed to learn spatiotemporal patterns [25]. Wei, Wong et al. [24] developed a 2D frame representation in which each pixel corresponded to an instantaneous value from an sEMG sensor electrode, and a multi-stream two-layer 2D CNN was used for gesture recognition. Related studies further showed that high-density sEMG, arranged into spatiotemporal grid frames, can be decoded using 3D CNNs for continuous hand kinematics [33]. By stacking high-density sEMG signal grids from forearm and wrist muscles into frame sequences, a 3D CNN was trained to predict finger joint angles during natural grasping, achieving a mean absolute error of 2.78 ± 0.28° and a mean correlation coefficient of 0.94. Although effective, these methods are mainly designed for high-density sEMG settings.

A relevant sparse-sensor example is the work of Wei, Li et al. [21], which extended the frame-based concept to continuous upper-limb kinematics estimation. Their method directly reconstructed multi-channel sEMG signals collected at each time point into 2D frames based on the anatomical distribution of target muscles and electrode placement. The frames were then stacked over a 200 ms sliding window to form a frame sequence, enabling a 3D CNN to learn spatial and temporal information directly from the frame sequence for wrist kinematics estimation. Their 3DCNN-BiLSTM model outperformed baseline 2DCNN-BiLSTM, 2DCNN, BiLSTM, and LSTM models using a direct 2D signal-image (channel × time) input per window, with reported average RMSE values of 0.14 and 0.04 on two datasets. This prior work shows that a CNN-based model using a frame-based representation can outperform its variants using a signal-image input. However, it relies on raw signal frames and a 3D CNN architecture, which increases input volume, memory footprint, and computational demand.

In frame-based representation pipelines, the temporal evolution is represented explicitly across frames, while the spatial arrangement is preserved within each frame. However, they can also become expensive without careful consideration of model development. For example, Wei, Wong et al. [24] used five parallel two-layer 2D CNN encoders to process sEMG frames, substantially increasing computational cost. In addition, 3D CNNs jointly model spatial and temporal dependencies from stacked frame sequences, but they generally require more memory than 2D CNNs. One study reported that a 3DCNN-based model with matched layer depth and filter counts required 60% more memory to store trainable parameters than the corresponding 2DCNN-based model [34]. In the case of Wei, Li et al. [21], the use of three parallel 3D CNN encoder branches and an attention-enhanced pipeline further increased architectural complexity and computational demand. Directly converting raw sEMG samples into a frame at every time step also enlarges the input volume and processing burden.

Therefore, our contribution is not the introduction of frame-based spatial mapping itself. Instead, we adapt the prior anatomy-informed frame-based sEMG mapping concept [21] into a feature-based frame representation, in which RMS features rather than raw sEMG samples are placed into sparse 2D frames, and we pair this representation with a lightweight 2D CNN encoder plus LSTM rather than a 3DCNN-based model. In other words, compared with the feature-image representation, the proposed input remains a frame sequence rather than a single generic 2D feature matrix. Compared with prior frame-based methods, it uses compact RMS feature frames rather than raw signal frames and a lighter spatial-temporal backbone targeted at quantization and embedded inference. In this way, the proposed model specifically targets lightweight and deployment-oriented knee moment estimation on resource-constrained devices, which has not been the focus of previous studies.

## 3. Materials and Methods

### 3.1. Dataset

For this study, we used a public dataset from Scherpereel et al. [35], which provides synchronized lower-limb biomechanics measurements along with wearable sensing signals across a wide range of cyclic and non-cyclic movements. Specifically, the dataset contains recordings from 12 healthy adults (7 males, 5 females, mean ± SD: age = 21.8 ± 3.2 years, height = 176.7 ± 8.6 cm, weight = 76.9 ± 14.4 kg) performing 11 cyclic activities (e.g., walking, running) and 20 non-cyclic activities (e.g., sit-to-stand, jumping, squatting, lunging, cutting), with lower limb joint kinematics based on a motion capture system and IMUs, joint kinetics from ground reaction forces, and sEMG. Motion capture data were collected at 200 Hz using a Vicon motion capture system. Ground reaction forces were measured using an instrumented treadmill and force plates at 1000 Hz, depending on the task. Force plate data were collected at 1000 Hz, and then low-pass filtered at 20 Hz using a 5th-order Butterworth zero-lag filter, and clamped to zero for forces less than 20 N. For data collection, the motion capture cameras were calibrated and verified to be within an acceptable error prior to data collection. In the case of a marker falling off during a trial, the marker was replaced and then a new static calibration was performed. sEMG electrodes were placed according to the SENIAM guidelines [36] and the orientation of the IMUs was verified by comparing the orientation of the gravity vector across participants during standing.

**Figure 1:**
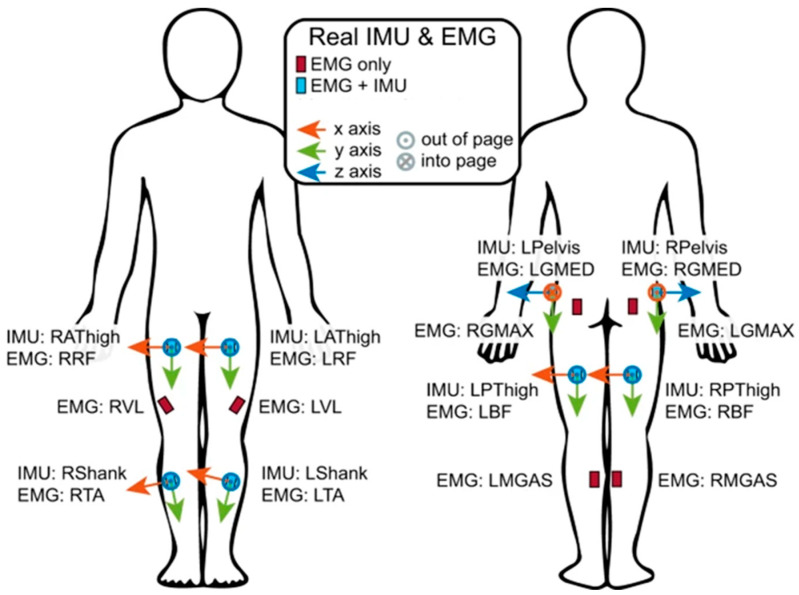
The orientations, placement, and axes definitions for physical inertial measurement units (IMUs) and surface electromyography sensors (sEMGs). Reprinted from the reference [35].

Subject-specific musculoskeletal models in OpenSim were scaled and used to compute inverse kinematics, followed by inverse dynamics. Hip, knee and ankle joint moments were normalized based on body mass. These moments were computed at 200 Hz and processed using a forward-reverse 5th-order Butterworth low-pass filter with a 6 Hz cut-off frequency. In Figure 1, wearable signals were collected using Delsys Trigno Avanti sensors. Bilateral sEMG data were recorded at 1259 Hz from the following muscles: gluteus medius, gluteus maximus, gracilis (GRAC), biceps femoris (BF), vastus lateralis (VL), rectus femoris (RF), tibialis anterior (TA), and medial gastrocnemius (MGAS). The sEMG signal was then upsampled to 2000 Hz. IMU sensors were placed on the gluteus medius, BF, RF, and TA. In the present study, we used only motion data from level-ground treadmill walking at two speeds: 1.2 and 1.8 m/s. These speeds represent typical outdoor walking conditions in healthy adults, aligning with reported usual walking speeds (1.27–1.35 m/s) and fast walking capabilities (1.45–1.79 m/s) [37]. The walking data for each subject lasted for 20 s. Since the dataset lacked the right knee flexion moment data, we used the filtered left knee flexion moment as the ground-truth target. After removing invalid data in one subject, data from 11 subjects were chosen for model training and testing. From the available sEMG channels, we selected five muscles relevant to knee moment generation as model inputs: knee extensors (VL, RF) and knee flexors (BF, GRAC and MGAS), as model inputs [11,38].

### 3.2. Work Pipeline

**Figure 2:**
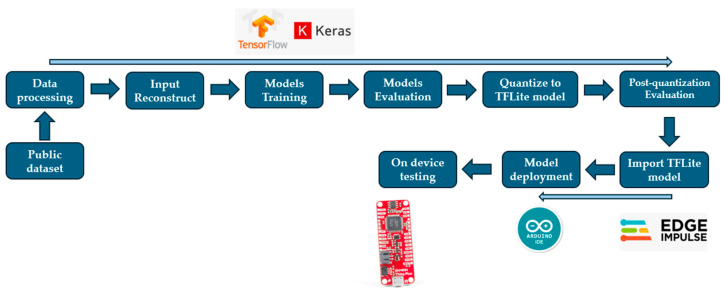
Workflow for model development, evaluation, and embedded deployment.

Figure 2 summarizes the workflow used in this study, from data preprocessing and input reconstruction through model training to deployment. The proposed Topo2DCNN-LSTM and all baseline models were first trained and evaluated before quantization. The trained networks were then quantized to TensorFlow Lite format. After conversion, the quantized models were re-evaluated to quantify any performance change due to quantization. Finally, the quantized Topo2DCNN-LSTM was deployed to the SparkFun Thing Plus–SAMD51 via Edge Impulse and Arduino for on-device model inference testing. The platforms and tools used at each stage are described in the following sections.

### 3.3. Data Preprocessing

To ensure data usability, comprehensive pre-processing was performed prior to training. The preprocessing pipeline consisted of filtering, RMS feature extraction, train/test splitting, sample-wise alignment with knee moment, sliding-window segmentation, and channel-wise normalization. Initially, raw sEMG signals from five channels were bandpass filtered using a zero-phase 4th-order Butterworth filter with a frequency range of 20–450 Hz [39], and then a zero-phase notch filter at a cutoff frequency of 50 Hz was applied to the signals to remove power-frequency noise [40].

Frequency-domain, time-domain, and time-frequency-domain features are widely used in sEMG signal processing [41]. Compared with frequency-domain and time-frequency-domain features, time-domain sEMG features can be extracted more quickly and achieve better real-time performance [11,20]. Accordingly, root mean square (RMS), a time-domain feature, was used to construct the model inputs, consistent with previous work on online joint angle prediction [11]. RMS was selected here as a compact representation that remained suitable for sequential frame construction and lightweight, resource-constrained inference. The RMS feature extraction formula is shown in (1).

(1) $$\mathrm{RMS}=\sqrt{\frac{1}{N}\sum_{n=1}^{N}EMG(n{)}^{2}}$$

Here, EMG(n) is the filtered sEMG sequence, n is the number of sEMG sequences, and N is the window width.

After filtering, the continuous sEMG signals sampled at 2000 Hz were divided, for each subject, into training and test segments using an 80%/20% split. RMS features were then extracted separately from the training and test segments at 5 ms intervals, resulting in five-channel RMS sequences sampled at 200 Hz. Because the ground-truth knee moment was also available at 200 Hz, the RMS features in each split were aligned sample by sample with the corresponding knee moment signal, without additional downsampling or resampling. To prevent information leakage, sliding-window segmentation was performed only after the train/test split, so that overlapping windows were generated independently within each split.

The window length and overlap were selected to balance predictive performance and estimation latency for the current dataset. To determine the most suitable window size, preliminary experiments were conducted using the training segments to train the baseline models across subjects with different window settings, as listed in Table 1. The range of window sizes was selected based on previous studies on lower-limb joint moment estimation using time-domain sEMG features [5,9]. The window setting that produced the best overall average baseline-model performance for this dataset, while remaining compatible with the latency requirement, was then used in all subsequent experiments. A 100 ms window with 70% overlap was applied to the RMS sequences, producing input samples with a size of 20 × 5 (time × channel) for each window. Z-score normalization was then performed per sensor channel using the training-set RMS statistics, and the same normalization parameters were applied to the test set.

All data filtering, feature generation, and segmentation were conducted in Python 3.11 [42].

### 3.4. Input Reconstruction

#### 3.4.1. Feature-Based Frame Construction

Motivated by prior sEMG feature-image studies and previously reported frame-based representation approaches, we constructed a feature-based sequential frame representation for knee moment estimation. Following the general frame-construction idea in [21], we used engineered sEMG features rather than raw per-sample sEMG amplitudes as the frame content. Specifically, each time point within a sliding window was represented by a five-channel RMS feature vector, and this vector was embedded into a fixed sparse 2D grid to form one frame. Consecutive frames within the window were then arranged as a frame sequence for subsequent spatial-temporal modelling.

During walking, the net knee joint moment arises from coordinated activation across antagonistic muscle groups. Among the selected channels, RF and VL contribute primarily to knee extension, whereas posterior and medial muscles such as BF, GRAC, and gastrocnemius can contribute to knee flexion and joint stabilization [38]. In the present study, this functional grouping was used only to guide a consistent frame arrangement across channels. It was not intended to represent a complete or optimal anatomical model of knee moment generation. Accordingly, the grid positions were assigned using the relative distribution of the selected muscle groups so that the same channel consistently occupied the same location across all frames. This placement served as a practical rule for constructing a reproducible 2D frame layout for sparse sEMG sensors [21]. The purpose of this design was to preserve a consistent inter-muscle arrangement for the model input.

**Figure 3:**
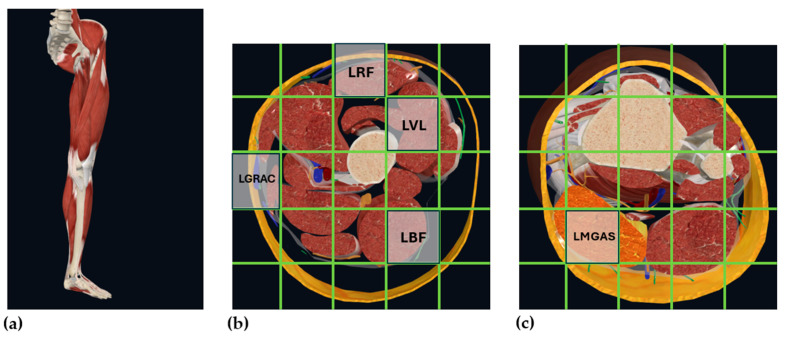
The isolated left leg musculoskeletal model (**a**), and the axial cross-section map of thigh (**b**) and lower leg (**c**) generated from the Complete Anatomy software (3D4Medical Ltd.), where LGRAC refers to gracilis in left thigh, LBF refers to biceps femoris in left thigh, LVL refers to vastus lateralis in left thigh, LRF refers to rectus femoris in left thigh, and LMGAS refers to medial gastrocnemius in left lower leg.

**Figure 4:**
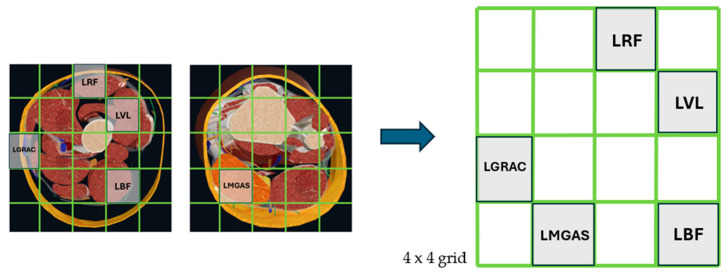
The mapping grid for RMS features of sEMG after reconstruction.

**Figure 5:**
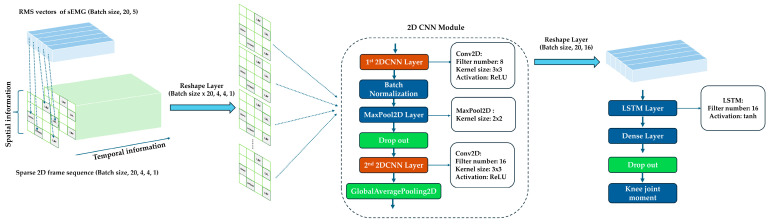
Topo2DCNN-LSTM model architecture and data flow pipeline.

To construct the frame, we referred to anatomical cross-section maps of the left thigh and lower leg generated from a commercial musculoskeletal model, as shown in Figure 3. Specifically, we located and placed the feature data based on the relative anatomical distribution of the five target muscles across the thigh and lower leg from the Complete Anatomy software 12.1.3.0 (3D4Medical Ltd., Dublin, Ireland). A 4 × 4 sparse grid was then constructed as a single 2D frame in Figure 4, with the empty cells set to zero. By stacking the 2D frames within the sliding window, a 2D frame sequence was obtained and fed into our Topo2DCNN-LSTM as model input, as shown in Figure 5. Because the present study focused on evaluating a lightweight deployment-oriented implementation, we did not perform ablation studies on alternative grid sizes, alternative placement rules, or sensitivity to electrode-location perturbations.

#### 3.4.2. RMS Sequence Input

For the TCN, LSTM and BiLSTM baselines, the input was the RMS feature sequence. Each sliding window was represented as a matrix of shape (20 × 5), where 20 is the number of RMS samples per window, and 5 is the number of channels. This representation preserves temporal structure and allows these models to learn within-window temporal dependencies directly.

#### 3.4.3. Time × Channel Feature-Image Input

For the 2DCNN-LSTM and 2DCNN-GRU baselines, the same RMS values were reshaped into a 2D image-like tensor of shape (20 × 5 × 1), where the additional dimension represents a single image channel for Conv2D processing. This feature-image representation allows the CNN-based baselines to process the RMS window as a feature image, while keeping the underlying RMS information the same as in the sequence-based input.

### 3.5. Model Architectures

#### 3.5.1. Proposed Topo2DCNN–LSTM

Because the input contains both spatial and temporal structure, we developed a hybrid deep learning model to process frame sequences efficiently. A 2D CNN encoder alone cannot model temporal evolution across a sequence of frames, whereas a 3D CNN encoder typically incurs higher computational cost and memory usage because the convolution kernels operate across both spatial and temporal dimensions. On resource-constrained edge devices, this can substantially increase peak RAM usage and inference latency. To address this issue, we developed a lightweight Topo2DCNN-LSTM.

The model pipeline is shown in Figure 5, and the architecture and parameter counts are summarized in Table 2. In this study, five-channel RMS feature vectors per window were reconstructed to a sequence of 2D anatomical frames as model input. To adapt 2D CNNs and LSTMs for sequence modelling, we used the TimeDistributed wrapper [43] and introduced a Reshape layer that flattened the time dimension into the batch dimension. Each input sequence of frames is represented as a five-dimensional tensor of shape (B, T, H, W, C), where B is the batch size, T = 20 is the number of frames per sliding window, H = W = 4 are the spatial dimensions of the grid, and C = 1 is the number of input channels. The reshape operation converted this tensor into a four-dimensional tensor of shape (B × 20, 4, 4, 1), allowing standard 2D convolutional layers to extract spatial features from each frame independently.

After reshaping, the 2D CNN module processed each frame independently using shared kernels across all time steps. In this way, the network learned a single spatial feature extractor that was applied uniformly to every frame in the sequence without increasing the number of parameters. Following a previous study [20], the CNN module consisted of two Conv2D layers with a kernel size of 3 × 3, which captured localized spatial patterns related to muscle activation and knee moment estimation. Each convolutional layer was followed by a rectified linear unit (ReLU) activation to introduce non-linearity. Because the input frames were small (4 × 4), a modest number of filters and a kernel size of 3 × 3 were sufficient. We therefore used a lightweight progression from 8 to 16 filters, which increased feature dimensionality in a controlled way with minimal computational overhead. To further improve training stability and reduce overfitting risk, a Batch Normalization layer and a dropout layer with a dropout rate of 20% were inserted between the two convolutional layers. A MaxPool2D layer was used to downsample the spatial resolution within each frame, and a GlobalAveragePooling2D layer was then applied to reduce dimensionality further so that each frame was represented by a feature vector of length 16. A second Reshape layer restored the time dimension, yielding a three-dimensional tensor of shape (B, 20, 16), which was then passed to the LSTM layer.

LSTM is well suited to regression on sequential data because it can model long-term temporal dependencies [44]. We selected 16 hidden units to balance model capacity for learning complex patterns with computational cost. The standard hyperbolic tangent (tanh) activation is used to bound internal states and outputs, helping maintain stable and consistent estimation. The output from the LSTM layer is then passed to a fully connected (Dense) layer, followed by a dropout layer with a dropout rate of 20% to reduce the effect of overfitting, and an output layer to produce one estimate per sliding window.

Overall, this design preserved the frame-based representation while keeping the input and intermediate tensors small, making the model suitable for embedded inference under limited memory and computation budgets.

#### 3.5.2. Baseline Models

To benchmark the proposed Topo2DCNN–LSTM, we compared it with several deep learning baselines commonly used in sEMG-based regression and classification. The selected baselines were chosen to represent major deep learning families under similar model complexity, including recurrent models (LSTM, BiLSTM), convolution-based temporal modelling (TCN), and hybrid convolutional–recurrent models (2DCNN-LSTM and 2DCNN-GRU). TCN was included because dilated temporal convolutions have shown efficient sequence-modelling capability in biosignal applications [19,45]. Given the small dataset, the 2DCNN-GRU baseline was included because GRU-based hybrids provide a lightweight alternative to CNN-LSTM and have shown better performance than CNN-LSTM in related sEMG studies [46,47]. Including both 2DCNN-LSTM and 2DCNN-GRU therefore enabled comparison of the proposed model against two closely related lightweight hybrid baselines with different recurrent units.

All models used the same five-channel RMS sliding-window dataset, identical pre-processing, and the same training settings, including loss, optimizer, batch size and epochs. Layer widths, including filter counts and hidden units, were chosen to keep model capacity in a similar range to the proposed network.

TCN: As for the baseline TCN model, it consists of a TCN block [19] for capturing temporal dependencies, followed by a GlobalAveragePooling1D layer, a dense layer, and a dropout layer. Within the TCN block, two Conv1D layers are used with dilation rates of 1, 2, and 4 to extract temporal features at multiple scales. The same 16 filters are applied across all Conv1D layers to maintain consistency and keep the model complexity similar to that of the proposed Topo2DCNN-LSTM. Additionally, two dropout layers are added within the TCN block to reduce overfitting. The model architecture details are shown in Table 3.

LSTM: As for the baseline LSTM model, it consists of two LSTM layers that capture temporal dependencies within the window, followed by a dense layer and a dropout layer. The same 16 hidden units are applied across the LSTM layers to maintain consistency. This baseline represents a standard recurrent approach for EMG time-series regression. Additionally, to minimise overfitting, a Batch Normalization layer has been added within the stacked LSTM layers. The model architecture details are shown in Table 4.

BiLSTM: The BiLSTM baseline used the same input shape as the LSTM baseline, but with bidirectional recurrent layers to incorporate both forward and reverse temporal context within the window [48]. The same 16 hidden units are applied across the BiLSTM layers to maintain consistency. Additionally, to minimize overfitting, a Batch Normalization layer has been added within the stacked BiLSTM layers. This baseline tests whether using the full window temporal context improves knee moment estimation under the same data and training conditions. The details of the model architecture are shown in Table 5.

2DCNN-LSTM: This baseline used a time × channel × 1 RMS feature image for each sliding window as input, as described in Section 3.4.3. It retained the same architecture as the Topo2DCNN-LSTM except that the GlobalAveragePooling2D layer was removed. Because the 2DCNN-LSTM input is a single 2D feature image rather than a sequence of frames, applying GlobalAveragePooling2D would collapse the temporal dimension into one feature vector and prevent subsequent LSTM-based temporal modeling. This baseline therefore served as a control while keeping the CNN-LSTM architecture broadly comparable. The detailed architecture is shown in Table 6.

2DCNN-GRU: This baseline used the same input format as the 2DCNN-LSTM and followed the same 2D CNN framework, with the LSTM layer replaced by one GRU layer [15] for temporal modelling. To maintain comparable model complexity, the GRU layer was configured with 16 hidden units. The detailed architecture is shown in Table 7.

### 3.6. Training Configuration

The whole model development was conducted on a system equipped with an Intel 13th Gen Core i7-13620H CPU, 16 GB of RAM, and Intel UHD Graphics with 8 GB. All models were trained for 40 epochs with a mini-batch size of 16 to ensure consistency. Mean squared error (MSE) was used as the loss function, and optimization was performed using the Adam optimizer implemented in TensorFlow [49], with a learning rate of 0.0005 and gradient clipping with a clip norm of 1.0.

Moreover, to enhance model stability and prevent overfitting, ReduceLROnPlateau was employed to reduce the learning rate by a factor of 0.5 when the validation loss failed to improve for six consecutive epochs. In addition, an EarlyStopping criterion was applied to terminate training if validation loss did not improve for 12 consecutive epochs, maintaining the best-performing model weights.

### 3.7. Evaluation Metrics and Statistical Analysis

Model performance on the walking test data was evaluated using two gait-cycle-based metrics: mean gait-cycle-based RMSE and mean gait-cycle-based *R*^2^. Gait events were identified from the ground reaction force data, and three consecutive complete gait cycles were extracted for each subject at each walking speed. For each extracted gait cycle, the RMSE and *R*^2^ between the predicted and ground-truth left knee moment trajectories were computed. The three cycle-specific RMSE and *R*^2^ values were then averaged to obtain the mean gait-cycle-based RMSE and *R*^2^ for that subject at each walking speed.

Before selecting the inferential framework, assumptions for parametric repeated-measures analysis were examined. Shapiro–Wilk tests on subject-level paired differences indicated that normality was not consistently satisfied in model comparisons. The corresponding sphericity diagnostics for the initially considered parametric framework are provided in Table A1, Table A2, Table A3, Table A4, Table A5 and Table A6 in Appendix A. Given the small sample size and these assumption concerns, appropriate statistical analysis tests for all models were performed using the Friedman test, followed by Holm-corrected Wilcoxon signed-rank post hoc comparisons [50]. The paired differences of the evaluation metric between the proposed model and all baselines were summarized using Hodges–Lehmann estimates (median difference) and 95% confidence intervals in Table A2, Table A3, Table A5 and Table A6 in Appendix A. Statistical significance was set at *p* < 0.05.

The evaluation metric definitions are shown below:

(2) $$RMSE=\sqrt{\frac{1}{N}\sum_{i=1}^{N}{\left(y_{i}-{\hat{y}}_{i}\right)}^{2}}$$

(3) $$R^{2}=1-\frac{\sum_{i=1}^{N}{\left(y_{i}-{\hat{y}}_{i}\right)}^{2}}{\sum_{i=1}^{N}{\left(y_{i}-\overset{-}{y}\right)}^{2}},\overset{-}{y}=\frac{1}{N}\sum_{i=1}^{N}y_{i}$$

where N, test samples, ground truth left knee moment in Nm/kg, $y_{i}$, and estimated left knee moment in Nm/kg, ${\hat{y}}_{i}$. A lower RMSE and an *R*^2^ value closer to 1 indicate better regression performance.

### 3.8. Quantization and Deployment

All models were implemented, trained, quantized, and exported using TensorFlow with the Keras API [51]. TensorFlow is an open-source deep learning framework that supports deployment across mobile, embedded, and edge platforms. To enable execution on resource-constrained hardware, the trained models were converted to TensorFlow Lite and then quantized using full-integer quantization. This method reduces computational cost and memory usage by converting both weights and activations to 8-bit integers (int8) [31]. To reduce accuracy loss during conversion, a representative dataset was required to calibrate the activation ranges. Therefore, 200 samples from the training set were used for calibration.

Edge Impulse is a cloud-based machine-learning operations platform for TinyML systems and provides a Bring Your Own Model (BYOM) mode that allows pre-trained models from external frameworks to be imported and deployed to edge devices [52]. After quantization, the int8 TensorFlow Lite models were imported into Edge Impulse. Edge Impulse Studio was then used to generate an Arduino-compatible C/C++ model library for embedded deployment.

In this study, the generated Arduino library was compiled and flashed to a SparkFun Thing Plus–SAMD51 MCU (SparkFun Electronics, Niwot, CO, USA) [53] (32-bit ARM Cortex-M4F, up to 120 MHz, 1 MB flash, and 256 kB internal SRAM). Edge Impulse Studio was used to simulate on-device performance and to obtain estimated RAM usage, flash usage, and inference latency for each model. To test the quantized model on the target MCU, we followed the implementation approach described in [54], in which a single buffered input sample was provided to the compiled model on the device for inference. This setup enabled inference testing without requiring live sEMG acquisition. Arduino IDE was then used to estimate on-device model inference latency and memory usage on the target MCU.

All models were quantized to int8 and re-evaluated because quantization can affect predictive accuracy across architectures. On-device deployment testing was performed only for the proposed model, because embedded inference feasibility was one of the main objectives of this study.

## 4. Results

### 4.1. Model Performance

**Figure 6:**
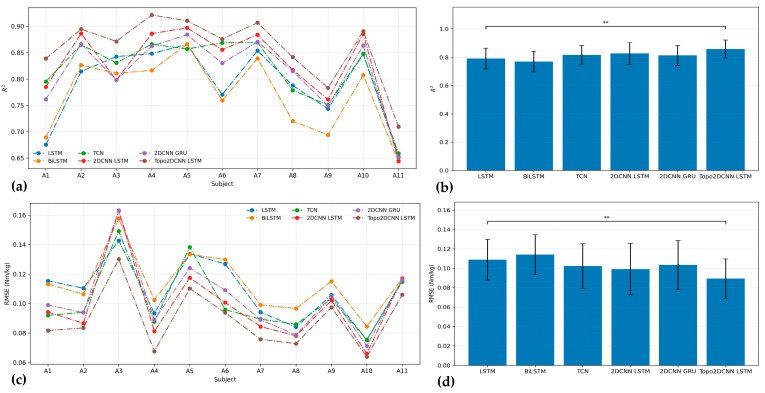
*R*^2^ (**a**) and RMSE (**c**) results for all models of 11 subjects. The performance for average *R*^2^ (**b**) and RMSE (**d**) with the corresponding standard deviations for each model at a walking speed of 1.2 m/s. The significance level is set as 0.05 (** *p* < 0.01).

**Figure 7:**
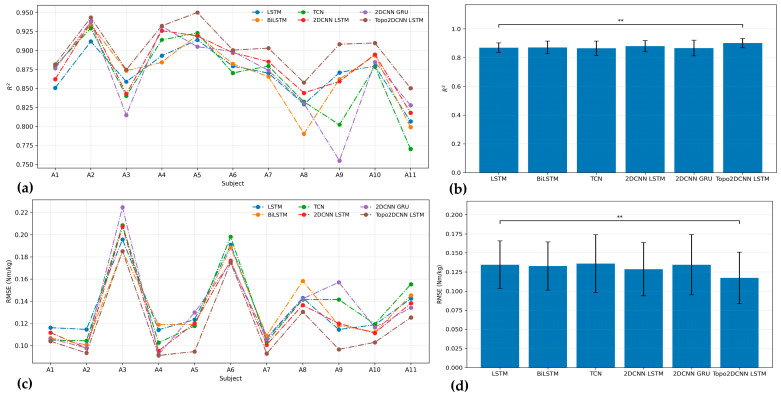
*R*^2^ (**a**) and RMSE (**c**) results for all models of 11 subjects. The performance for average *R*^2^ (**b**) and RMSE (**d**) with the corresponding standard deviations for each model at a walking speed of 1.8 m/s. The significance level is set as 0.05 (** *p* < 0.01).

Figure 6 and Figure 7 and Table 8 summarize the pre-quantization performance of the proposed Topo2DCNN-LSTM and the five baseline models at walking speeds of 1.2 and 1.8 m/s. At both speeds, the proposed model achieved the best average performance across all evaluation metrics. At 1.2 m/s, it reached an average *R*^2^ of 0.858 ± 0.063 and a mean RMSE of 0.087 ± 0.019 Nm/kg. At 1.8 m/s, the corresponding values were *R*^2^ = 0.900 ± 0.033 and mean RMSE = 0.113 ± 0.034 Nm/kg. The strongest baseline overall was 2DCNN-LSTM, which achieved *R*^2^ = 0.829 ± 0.078 and RMSE = 0.096 ± 0.024 Nm/kg at 1.2 m/s, and *R*^2^ = 0.881 ± 0.038 and RMSE = 0.123 ± 0.034 Nm/kg at 1.8 m/s. Although the subject-level differences for 11 subjects were generally modest in Figure 6 and Figure 7, the proposed model showed a consistent overall shift toward better performance. This advantage was more evident at the lower walking speed, where Friedman effect sizes ranged from 0.634 to 0.686, compared to 0.504 at 1.8 m/s, indicating a stronger overall model effect at 1.2 m/s.

We reported the statistical comparison between the proposed model and the 2DCNN-LSTM baseline because both models share the same CNN–LSTM backbone and identical trainable parameter count (3657) as shown in Table 2 and Table 6. The only difference was the input representation. As shown in Table 9, the proposed model achieved higher *R*^2^ and lower errors at both walking speeds, with median improvements of Δ*R*^2^ = 0.029 and ΔRMSE = −0.009 Nm/kg at 1.2 m/s, and Δ*R*^2^ = 0.019 and ΔRMSE = −0.009 Nm/kg at 1.8 m/s. These differences indicate a modest but consistent subject-level advantage, which translated into a clearer improvement in the averaged results. The corresponding absolute effect size was 0.885 for all metrics, indicating a strong matched-baseline effect.

**Figure 8:**
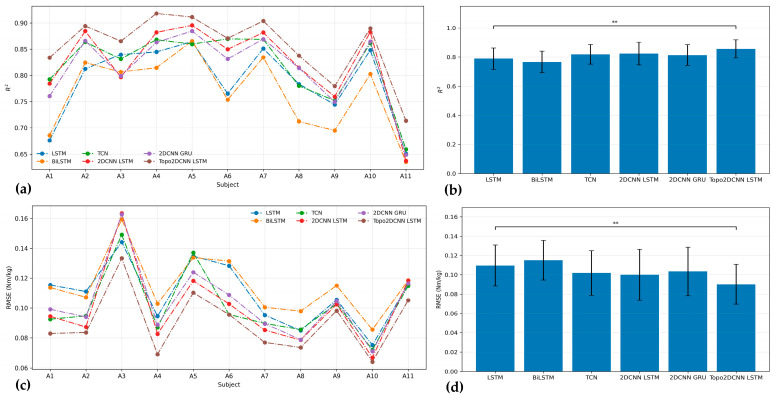
*R*^2^ (**a**) and RMSE (**c**) results for all int8 models of 11 subjects. The performance for average *R*^2^ (**b**) and RMSE (**d**) with the corresponding standard deviations for each model at a walking speed of 1.2 m/s. The significance level is set as 0.05 (** *p* < 0.01).

**Figure 9:**
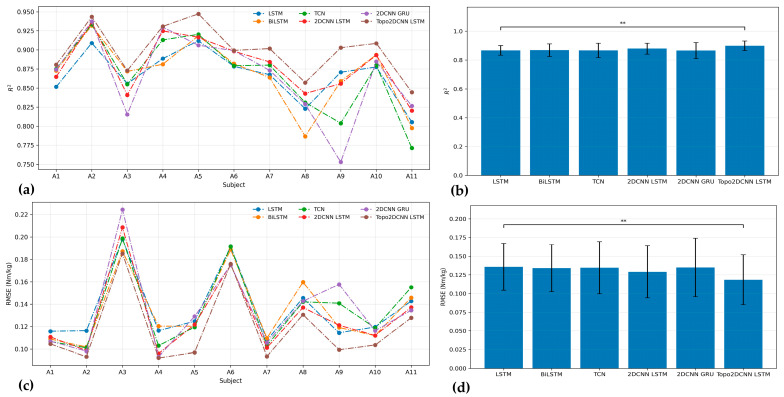
*R*^2^ (**a**) and RMSE (**c**) results for all int8 models across 11 subjects and model performance for average *R*^2^ (**b**) and RMSE (**d**) with the corresponding standard deviations for each model at a walking speed of 1.8 m/s. The significance level is set as 0.05 (** *p* < 0.01).

Figure 8 and Figure 9 and Table 10 summarize the results after full int8 quantization. Quantization produced only a small decrease in average performance, and the proposed model remained the best-performing method across both walking speeds. After quantization, Topo2DCNN-LSTM achieved *R*^2^ = 0.856 ± 0.062 and RMSE = 0.088 ± 0.020 Nm/kg at 1.2 m/s, and *R*^2^ = 0.898 ± 0.034 and RMSE = 0.114 ± 0.034 Nm/kg at 1.8 m/s. Similar to the floating-point results, the subject-level differences after quantization remained relatively small, but the proposed model preserved a consistent average advantage over the 2DCNN-LSTM baseline. Table 10 also presents the Friedman test effect sizes for the int8 models. At 1.2 m/s, the effect sizes were 0.677 for *R*^2^ and 0.685 for RMSE, indicating a relatively strong overall model effect. At 1.8 m/s, the corresponding effect sizes were 0.497 for *R*^2^ and 0.546 for RMSE, suggesting a moderate overall difference among models that was smaller than that observed at 1.2 m/s. In Table 11, the modest median improvements after quantization remained close to those of the floating-point model, with Δ*R*^2^ = 0.028 and ΔRMSE = −0.009 Nm/kg at 1.2 m/s, and Δ*R*^2^ = 0.018 and ΔRMSE = −0.008 Nm/kg at 1.8 m/s. The associated absolute effect sizes remained high, indicating that the advantage of the proposed model was preserved after model compression.

**Figure 10:**
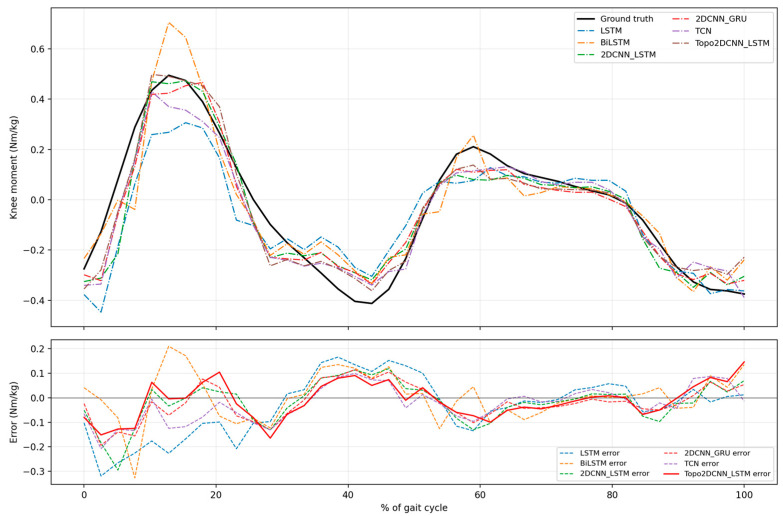
Examples of the model performance comparison between average actual and estimated normalized knee joint moment for subject A2 at a walking speed of 1.2 m/s. The error differences are based on all int8 models.

**Figure 11:**
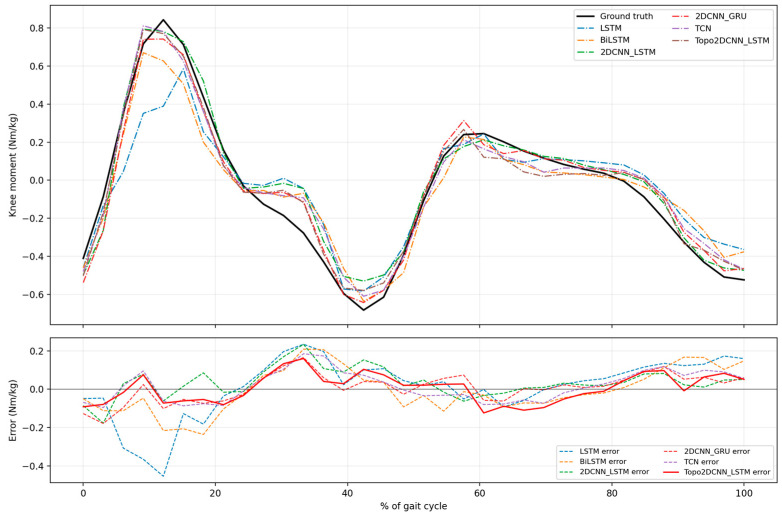
Examples of the model performance comparison between average actual and estimated normalized knee joint moment for Subject A2 at a walking speed of 1.8 m/s. The error differences are based on all int8 models.

**Figure 12:**
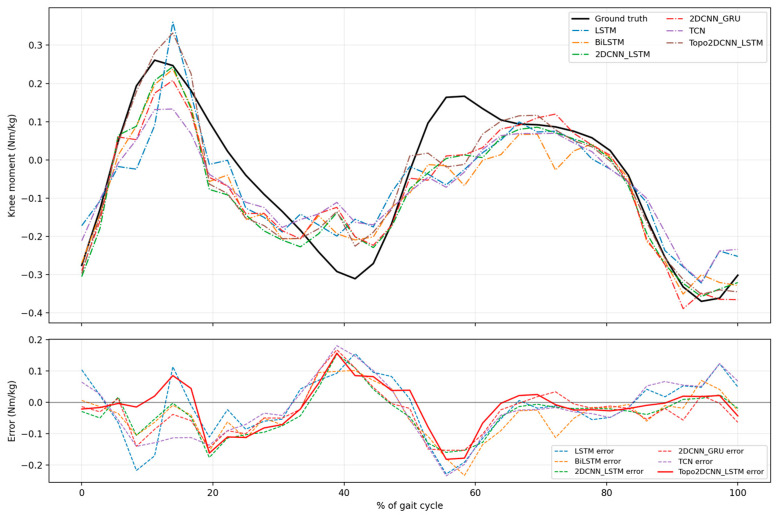
Examples of the model performance comparison between average actual and estimated normalized knee joint moment for Subject A11 at a walking speed of 1.2 m/s. The error differences are based on all int8 models.

**Figure 13:**
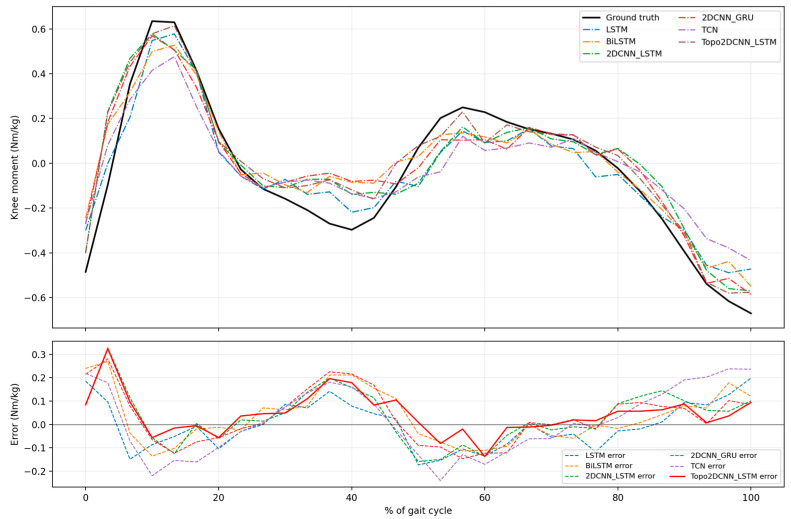
Examples of the model performance comparison between average actual and estimated normalized knee joint moment for Subject A11 at a walking speed of 1.8 m/s. The error differences are based on all int8 models.

Figure 10, Figure 11, Figure 12 and Figure 13 present qualitative examples of the mean gait-cycle overlap between the measured knee moment and the estimated knee moments from all int8 models for two representative subjects at both walking speeds. Subject A2 was selected as a representative higher-performing subject because, as shown in Figure 8 and Figure 9, it generally exhibited higher *R*^2^ values and lower RMSE values across models at both 1.2 and 1.8 m/s. In contrast, subject A11 was selected as a representative lower-performing subject because its estimation performance was generally weaker across both walking speeds. Figure 10 and Figure 11 compare the measured knee moment with the estimated knee moments from the proposed Topo2DCNN-LSTM and the five baseline models for subject A2 at 1.2 and 1.8 m/s, respectively. In the upper panel, the black curve denotes the measured knee moment, whereas the lower panel shows the prediction error for each model; smaller absolute error indicates better agreement with the measured waveform. At 1.2 m/s in Figure 10, the proposed model follows the waveform closely and shows the clearest error reduction relative to LSTM and BiLSTM, particularly around the first positive knee-moment peak at approximately 10% to 20% of the gait cycle and the terminal-stance valley at approximately 35% to 45% of the gait cycle. In this example, LSTM markedly underestimates the early peak, whereas BiLSTM shows a noticeable overshoot in the same region. Compared with TCN, 2DCNN-GRU, and 2DCNN-LSTM, the proposed model also shows lower error in these phases, although the differences are more modest. The main residual error for all models remains around the terminal-stance valley, where the depth of the negative moment is not fully captured. At 1.8 m/s in Figure 11, the estimated trajectories are more closely grouped, consistent with the smaller average performance differences at this speed shown in Figure 9. Even so, the proposed model still shows clearer error reduction than LSTM and BiLSTM at approximately 5% to 20% and 25% to 35% of the gait cycle, whereas its advantage over TCN, 2DCNN-GRU, and 2DCNN-LSTM is more modest and mainly visible around 25% to 35% of the gait cycle.

At 1.2 m/s in Figure 12, all models capture the overall waveform for subject A11, but larger inconsistencies are observed from mid-stance to pre-swing, approximately 10% to 60% of the gait cycle. In this example, the proposed model follows the waveform more closely, with smaller errors during loading response and the late portion of the gait cycle, whereas 2DCNN-GRU, LSTM, and TCN show more variable deviations, particularly around 0% to 20% and 80% to 100% of the gait cycle. At 1.8 m/s in Figure 13, the proposed model captures both the initial positive peak and the subsequent secondary positive peak reasonably well. However, all models tend to overestimate the moment at approximately 5% and 40% of the gait cycle, and to underestimate it at around 10% to 15%, corresponding to the end of loading response and the beginning of mid-stance, as well as near the second peak around 60% of the gait cycle. Within these challenging regions, the proposed Topo2DCNN-LSTM shows the smallest error near the main first positive moment peak at approximately 10% to 15% of the gait cycle and around the second positive peak region at approximately 50% to 65% of the gait cycle. Overall, these examples show that the proposed model preserves the main gait-cycle waveform in both a relatively higher-performing subject (A2) and a more difficult subject (A11), although the remaining errors remain concentrated around the positive moment peaks and the mid-stance to terminal-stance regions, which appear challenging for all compared models.

### 4.2. Model Deployment on the Microcontroller

In this study, we deployed the proposed Topo2DCNN-LSTM model on the MCU. Table 12 shows the memory usage and inference latency estimated by Edge Impulse for the quantized model. In contrast, Table 13 shows the average on-device RAM, flash, and latency estimates from the Arduino IDE. Since our sliding window length is 100 ms and the overlap percentage is 70%, the estimated inference time based on on-device testing was below the 30 ms latency target, demonstrating the feasibility of local model inference. However, the deployment result should be interpreted as an inference-feasibility test only, because live signal acquisition, filtering, RMS generation, buffering, and frame construction were not yet included in the end-to-end embedded pipeline.

## 5. Discussion

This study evaluated whether a lightweight 2DCNN-LSTM using a feature-based sequential frame representation could improve personalized knee moment estimation from sEMG under limited walking conditions while remaining suitable for isolated on-device inference. Across both walking speeds, the proposed Topo2DCNN-LSTM achieved the best average performance before and after full int8 quantization. Before quantization, it reached an average *R*^2^ of 0.858 ± 0.063 and RMSE of 0.087 ± 0.019 Nm/kg at 1.2 m/s, and *R*^2^ of 0.900 ± 0.033 and RMSE of 0.113 ± 0.034 Nm/kg at 1.8 m/s, as shown in Table 8. After quantization, only minor performance loss was observed, with *R*^2^ = 0.856 ± 0.062 and RMSE = 0.088 ± 0.020 Nm/kg at 1.2 m/s, and *R*^2^ = 0.898 ± 0.034 and RMSE = 0.114 ± 0.034 Nm/kg at 1.8 m/s, as shown in Table 10. Overall, the proposed model remained the best-performing method after model compression.

Across all models, average RMSE values were lower at 1.2 m/s than at 1.8 m/s. One possible explanation is that subjects may have adjusted their gait and balance more consciously at the faster and less familiar walking speed, leading to greater variability in motor control and muscle activation patterns. The overall advantage of the proposed model was also stronger at 1.2 m/s, as reflected by the larger Friedman effect sizes at the lower speed. This suggests that the clearest benefit of the proposed model was observed under the more typical walking condition, although it still remained the best-performing model at the faster walking speed.

A broader comparison across baselines suggests that combining spatial feature extraction with temporal modelling is beneficial. As shown in Table 8, the benefit of combining convolutional feature extraction with temporal modelling was more evident at 1.2 m/s, where 2DCNN-LSTM, 2DCNN-GRU, and TCN outperformed LSTM and BiLSTM in *R*^2^ and RMSE-based metrics. This trend is consistent with prior findings [21,55]. At 1.8 m/s, the clearest advantage over the recurrent-only baselines was observed for 2DCNN-LSTM, indicating that local cross-channel feature extraction provides useful information beyond temporal sequence learning alone.

The comparison between Topo2DCNN-LSTM and 2DCNN-LSTM was conducted because the two models share the same backbone and identical trainable parameter count (3657), as shown in Table 2 and Table 6, and differ mainly in their input representation. As shown in Table 9, the Topo2DCNN-LSTM showed median improvements of Δ*R*^2^ = 0.029 and ΔRMSE = −0.009 Nm/kg (9.4% lower) at 1.2 m/s, and Δ*R*^2^ = 0.019 and ΔRMSE = −0.009 Nm/kg (7.3% lower) at 1.8 m/s. These differences are modest in absolute magnitude, but they are consistent across metrics and walking speeds, which supports the interpretation that the representation choice contributes to performance beyond model complexity alone. This modest margin is expected, because the matched 2DCNN-LSTM baseline already used the same RMS features and a similar 2DCNN–LSTM backbone under identical model capacity. Therefore, the present comparison isolates a subtle representation effect rather than a broader architectural effect. Additionally, among the hybrid baselines, 2DCNN-LSTM outperformed 2DCNN-GRU in the present dataset as shown in Table 8 and Table 10. This result differs from some previous studies in which CNN-GRU slightly outperformed CNN-LSTM, but those studies were mainly focused on classification rather than continuous waveform regression [46,47]. One possible reason is that the LSTM provides a more effective memory mechanism for preserving temporal information relevant to continuous regression under the present windowed setting. In the present study, the lower parameter count of GRU did not translate into better estimation accuracy in this dataset, although it remains a reasonable lightweight baseline for deployment-oriented comparison.

By contrast, the advantage of the proposed Topo2DCNN-LSTM over 2DCNN-LSTM is more likely to reflect the effect of input representation than the choice of recurrent unit alone. A plausible explanation for this advantage is that the time × channel feature-image baseline does not represent a true spatial field, because one axis represents time progression while the other corresponds to sensor channels. As a result, 2D convolution in that format mixes temporal adjacency and channel adjacency within the same receptive field. In contrast, the feature-based sequential frame representation preserves a fixed channel arrangement within each frame and models temporal evolution explicitly across the frame sequence. This means that the convolutional encoder processes each time point as a consistent spatial pattern before the LSTM models frame-to-frame dynamics. The consistent improvement in *R*^2^ and RMSE suggests that the feature-based sequential frame representation supports better recovery of knee moment waveform shape. However, this interpretation remains indirect, because the present study did not include saliency maps, feature-attribution analysis, model architecture design ablation or controlled representation ablation experiments. These analyses remain important directions for future work.

Compared with Wei et al. [21], the present study does not propose frame-based spatial mapping itself but adapts that idea into a lighter and more deployment-oriented framework for a different biomechanical task. Wei et al. mapped raw multi-channel sEMG samples into anatomy-based 2D frames for forearm muscles and stacked them over time for a 3D CNN-based model to estimate continuous wrist kinematics. In contrast, the present study extends the same general concept to the left lower limb for sagittal-plane knee flexion moment estimation by embedding sparse multi-channel RMS features into fixed 2D frames and modelling the resulting frame sequence with a compact 2D CNN encoder and LSTM. Their architecture also combined three parallel 3D CNN encoder branches with BiLSTM and multi-head attention, which dramatically increases model complexity and computational demand [56]. By contrast, the present study used a single compact 2D CNN encoder with LSTM to better support lightweight deployment. In this way, the present framework retains the frame-based idea while using a compact feature-frame representation and a lightweight model architecture that remains suitable for quantization and microcontroller inference, while still showing consistent gains over the baselines under similar model complexity. Direct performance comparison is not appropriate because the two studies address different joints, outputs, datasets, and evaluation metrics.

To better understand how the observed performance gains varied across the gait cycle, subject-specific waveform examples were examined for A2 and A11. For subject A2, the proposed model showed the clearest error reduction around the early positive peak and terminal-stance region. At 1.2 m/s, the gain was most evident relative to LSTM and BiLSTM at approximately 10% to 20% and 35% to 45% of the gait cycle, while the advantage over TCN, 2DCNN-GRU, and the strongest baseline 2DCNN-LSTM was smaller but generally present in the same phases. At 1.8 m/s, the proposed model still showed lower error than LSTM and BiLSTM at approximately 5% to 20% and 25% to 35% of the gait cycle, whereas the difference relative to TCN, 2DCNN-GRU, and 2DCNN-LSTM was modest and mainly visible at approximately 25% to 35% of the gait cycle. For subject A11, the improvement pattern differed. At 1.2 m/s, the proposed model more closely followed the waveform at approximately 0% to 20% and 80% to 100% of the gait cycle, whereas 2DCNN-GRU, LSTM, and TCN showed larger deviations. At 1.8 m/s, the proposed model showed the smallest error at approximately 10% to 15% and 50% to 65% of the gait cycle. Together, these examples suggest that the benefit of the proposed model was dependent on both gait phase and subject, likely reflecting inter-subject variability in sEMG activation patterns and gait mechanics in the present personalized modelling setting [57]. Nevertheless, positive moment peaks and the mid-stance to terminal-stance phases remained challenging for all models.

From a biomechanical perspective, the error range observed in this study appears broadly comparable to that reported in previous walking-related joint moment estimation studies. The proposed model achieved mean gait-cycle-based RMSE values of 0.087 ± 0.019 Nm/kg at 1.2 m/s and 0.113 ± 0.034 Nm/kg at 1.8 m/s, which lie within the range reported in prior lower-limb joint moment estimation studies during walking. For example, Wang et al. [48] reported knee joint moment RMSE values of 0.16 ± 0.04 Nm/kg during treadmill walking using a TCN-BiLSTM network with three inertial measurement units. Xie et al. [58] reported RMSE values of approximately 0.13 Nm/kg for knee moment estimation across three walking speeds using sEMG and a transfer-learning-enhanced 2DCNN-GRU-Attention model. Similarly, Molinaro et al. [59] reported a knee joint moment RMSE of 0.13 Nm/kg across multiple walking conditions in an exoskeleton-oriented estimation framework based on a TCN model. However, making a fair comparison was difficult because the present study used a within-subject validation setting, whereas the previous studies adopted subject-independent or transfer-learning protocols with different sensor inputs and evaluation conditions. The present results should therefore be interpreted within the proof-of-concept scope of this study, noting that joint-moment error levels may vary with the task and population [60]. Although comparison with prior studies supports the biomechanical relevance of the observed error range, clinical meaningfulness was not evaluated directly because no application-specific clinical error threshold or patient-outcome analysis was included. Accordingly, more standardized benchmarking will be required in future work to support fairer cross-study comparison.

After full integer quantization, the proposed model retained essentially the same ranking and very similar performance margins over the baselines, indicating that the learned representation was largely preserved after compression. As shown in Table 10, quantization caused only minimal changes in the proposed model, with a reduction of 0.001 in *R*^2^ and a difference of 0.001 Nm/kg in RMSE at the two walking speeds. From a deployment perspective, the Edge Impulse EON™ Compiler estimated that the quantized model required 64.7 kB of RAM, 171.0 kB of flash, and 19 ms inference latency as shown in Table 12. In Table 13, when compiled and deployed on the SparkFun Thing Plus–SAMD51, the model used 71,316 bytes of RAM and 257,172 bytes of flash, with an average inference latency of 28 ms, as measured in the Arduino IDE. The increase in memory usage and latency is likely due to additional code dependencies on the target MCU. Under the present buffering scheme, this isolated inference time is within the 30 ms estimation update interval defined by the 100 ms window and 70% overlap, supporting the feasibility of isolated on-device inference for the quantized model. However, this should not be interpreted as full end-to-end real-time validation, because live acquisition, filtering, RMS feature extraction, buffering, and frame construction were not yet included in the embedded pipeline. This distinction is important because timing requirements in assistive systems depend on the complete control loop. For example, Molinaro et al. [59] reported that, for knee assistance, a 50 ms delay was the minimum achievable delay needed to maintain a consistent relationship between biological joint moment estimates and exoskeleton assistance, owing to loop-rate reliability limitations of the exoskeleton system. Therefore, while the present results support isolated inference feasibility, full embedded and closed-loop latency validation remains necessary in future work.

Overall, the present findings support the proposed framework as a proof of concept for personalized unilateral sagittal-plane knee flexion moment estimation under resource-constrained inference and limited walking conditions.

## 6. Limitations and Future Directions

While this study establishes an appropriate proof-of-concept framework for lightweight unilateral knee flexion moment estimation with isolated on-device inference, several factors should be considered when interpreting the results.

The current evaluation focused on treadmill walking at fixed speeds in a healthy-subject dataset. While these results demonstrate the fundamental capability of the proposed framework, performance may vary during activities with different gait patterns, such as stair negotiation or weighted walking. Future research should evaluate the robustness of this framework in larger, more diverse populations and a broader range of real-world conditions, including non-standardized scenarios such as sensor dropout. Additionally, future work should also investigate different temporal window lengths and alternative RMS feature-generation settings, as the optimal configuration may depend on movement dynamics. For example, a previous study of running gait analysis used a moving RMS window of 50 ms [61].

The present validation used an intra-subject split, which successfully supports the development of personalized models. While this does not yet establish cross-session or cross-subject generalization, it confirms the effectiveness of within-subject estimation under limited walking conditions. This approach accounts for individual differences in EMG amplitude, muscle coordination [57], and anatomy. Accordingly, the model’s performance in a cross-subject setting cannot be inferred from the present results. Future work can build on this by testing leave-one-subject-out protocols and explore lightweight calibration strategies, such as short offline segments or online standardization, to make the system more practical for daily use. These include an offline maximum voluntary contraction-based normalization or online standardization approaches based on saved subject-specific RMS statistics [11].

The present study focused on the left knee sagittal-plane flexion moment as a primary model output. This specific focus allowed for a clear demonstration of the Topo2DCNN-LSTM framework. Subsequent research can now extend this approach to other lower-limb joints, bilateral estimation, and more diverse movement tasks. Such extensions would help determine whether the proposed representation remains effective beyond the present single-output setting.

This research prioritized a deployment-oriented design over a complex, less portable architecture. While feature-visualization or saliency-based analysis was not included, the current results provide indirect evidence that the sequential frame representation captures meaningful features. A direct comparison between the present RMS-based representation and raw-sEMG inputs was outside the scope of this study. Future work can examine this under embedded-inference constraints. Furthermore, exploring lightweight attention-based mechanisms [62] and conventional machine-learning baselines will help ensure the most efficient balance between accuracy and resource usage is achieved. In addition, examining alternative frame layouts, grid resolutions, channel-assignment strategies, and reduced sensor-channel configurations may provide further insight into the robustness of the proposed representation.

The deployment tests focused on quantized model inference and memory usage, confirming that isolated on-device inference is feasible. This provides a foundation for future work to implement the full embedded signal-processing pipeline, including real-time acquisition and power evaluation. Moving toward end-to-end testing will be the important next step in transitioning this research from a laboratory proof of concept to a practical wearable solution. Future work should also define task-specific performance targets for rehabilitation monitoring and assistive-control applications, including acceptable RMSE ranges for practical use.

## 7. Conclusions

Building on a previously reported sEMG frame-construction concept, this study developed a lightweight 2DCNN-LSTM using a feature-based sequential frame representation for unilateral sagittal-plane knee flexion moment estimation and examined its feasibility for isolated on-device inference. Specifically, the present work adapted the prior frame-based concept from raw sEMG samples to compact RMS feature frames and paired it with a lightweight 2D CNN encoder and LSTM, rather than a 3DCNN-based architecture.

In intra-subject evaluation on a public walking dataset, the proposed model achieved the best average performance across the two treadmill walking speeds among the compared baselines (TCN, LSTM, BiLSTM, 2DCNN-LSTM, and 2DCNN-GRU). The proposed model reached *R*^2^ of 0.858 ± 0.063 and RMSE of 0.087 ± 0.019 Nm/kg at 1.2 m/s, and *R*^2^ of 0.900 ± 0.033 and RMSE of 0.113 ± 0.034 Nm/kg at 1.8 m/s. After full integer quantization, only minor performance loss was observed, and the same ranking was preserved. Specifically, it reached *R*^2^ = 0.856 ± 0.062 and RMSE = 0.088 ± 0.020 Nm/kg at 1.2 m/s, and *R*^2^ = 0.898 ± 0.034 and RMSE = 0.114 ± 0.034 Nm/kg at 1.8 m/s. The proposed model also showed modest but consistent gains over the feature-image 2DCNN-LSTM baseline after quantization, with pairwise median improvements of Δ*R*^2^ = 0.028 and ΔRMSE = −0.009 at 1.2 m/s, and Δ*R*^2^ = 0.018 and ΔRMSE = −0.008 at 1.8 m/s.

On the SparkFun Thing Plus–SAMD51, the quantized model required 71,316 bytes of RAM, 257,172 bytes of flash, and 28 ms average inference time, supporting the feasibility of isolated on-device inference. Overall, these findings support the proposed approach as a proof of concept for personalized unilateral sagittal-plane knee flexion moment estimation under resource-constrained inference and limited walking conditions in healthy subjects. Future work should evaluate cross-subject generalization, robustness to sensor-placement variability and broader walking conditions, the frame-construction design, and full end-to-end embedded implementation.

## Figures and Tables

**Table 1:** Window size and overlap percentage configuration.

Window Size (in ms)	100	150	200
	30%	30%	30%
	50%	50%	50%
	70%	70%	70%

**Table 2:** Topo2DCNN-LSTM model summary.

Model Layers	Parameters
Input layer	0
Reshape layer	0
Conv2D Layer 1	72
Batch Normalization Layer	16
MaxPool2D Layer	0
Dropout Layer	0
Conv2D Layer 2	1168
GlobalAvgPool2D Layer	0
Reshape layer	0
LSTM Layer	2112
Dense Layer	272
Dropout Layer	0
Output	17
Total trainable parameters	3657

**Table 3:** TCN model summary.

Model Layers	Parameters
Input layer	0
TCN Block	4272
GlobalAveragePooling1D	0
Dense Layer	272
Dropout Layer	0
Output	17
Total trainable parameters	4561

**Table 4:** LSTM model summary.

Model Layers	Parameters
Input layer	0
LSTM Layer	1408
Batch Normalization Layer	32
LSTM Layer	2112
Dense Layer	272
Dropout Layer	0
Output	17
Total trainable parameters	3841

**Table 5:** BiLSTM model summary.

Model Layers	Parameters
Input layer	0
Bidirectional (LSTM Layer)	2816
Batch Normalization Layer	128
Bidirectional (LSTM Layer)	6272
Dense Layer	528
Dropout Layer	0
Output	17
Total trainable parameters	9697

**Table 6:** 2DCNN-LSTM model summary.

Model Layers	Parameters
Input layer	0
Reshape layer	0
Conv2D Layer 1	72
Batch Normalization Layer	16
MaxPool2D Layer	0
Dropout Layer	0
Conv2D Layer 2	1168
Reshape layer	0
LSTM Layer	2112
Dense Layer	272
Dropout Layer	0
Output	17
Total trainable parameters	3657

**Table 7:** 2DCNN-GRU model summary.

Model Layers	Parameters
Input layer	0
Reshape layer	0
Conv2D Layer 1	72
Batch Normalization Layer	16
MaxPool2D Layer	0
Dropout Layer	0
Conv2D Layer 2	1168
Reshape layer	0
GRU Layer	1632
Dense Layer	272
Dropout Layer	0
Output	17
Total trainable parameters	3177

**Table 8:** Statistical analysis of all models at 1.2 and 1.8 m/s walking speeds.

Walking Condition	Model	*R*^2^ Mean ± Std	RMSE Mean ± Std (Nm/kg)
1.2 m/s	Topo2DCNN-LSTM	0.858 ± 0.063	0.087 ± 0.019
	2DCNN-LSTM	0.829 ± 0.078	0.096 ± 0.024
	2DCNN-GRU	0.814 ± 0.071	0.101 ± 0.022
	LSTM	0.791 ± 0.074	0.105 ± 0.019
	BiLSTM	0.770 ± 0.073	0.111 ± 0.017
	TCN	0.818 ± 0.066	0.100 ± 0.021
	Friedman test effect size	0.686	0.634
1.8 m/s	Topo2DCNN-LSTM	0.900 ± 0.033	0.113 ± 0.034
	2DCNN-LSTM	0.881 ± 0.038	0.123 ± 0.034
	2DCNN-GRU	0.867 ± 0.055	0.128 ± 0.038
	LSTM	0.869 ± 0.032	0.128 ± 0.032
	BiLSTM	0.873 ± 0.044	0.126 ± 0.031
	TCN	0.867 ± 0.051	0.129 ± 0.039
	Friedman test effect size	0.504	0.504

**Table 9:** Pairwise comparison between Topo2DCNN-LSTM and 2DCNN-LSTM at 1.2 and 1.8 m/s walking speeds.

Walking Condition	Metric	Median Difference [95%CI]	Absolute Effect Size
		Topo2DCNN-LSTM vs. 2DCNN-LSTM	
1.2 m/s	*R* ^2^	0.029 [0.015, 0.047]	0.885
	RMSE	−0.009 [−0.014, −0.006]	0.885
1.8 m/s	*R* ^2^	0.019 [0.011, 0.031]	0.885
	RMSE	−0.009 [−0.016, −0.006]	0.885

**Table 10:** Statistical analysis of all int8 models at 1.2 and 1.8 m/s walking speeds.

Walking Condition	Model	*R*^2^ Mean ± Std	RMSE Mean ± Std (Nm/kg)
1.2 m/s	Topo2DCNN-LSTM	0.856 ± 0.062	0.088 ± 0.020
	2DCNN-LSTM	0.826 ± 0.079	0.096 ± 0.024
	2DCNN-GRU	0.814 ± 0.071	0.101 ± 0.022
	LSTM	0.789 ± 0.073	0.106 ± 0.019
	BiLSTM	0.766 ± 0.074	0.112 ± 0.017
	TCN	0.820 ± 0.066	0.099 ± 0.021
	Friedman test effect size	0.677	0.685
1.8 m/s	Topo2DCNN-LSTM	0.898 ± 0.034	0.114 ± 0.034
	2DCNN-LSTM	0.880 ± 0.038	0.123 ± 0.034
	2DCNN-GRU	0.866 ± 0.056	0.129 ± 0.038
	LSTM	0.867 ± 0.033	0.129 ± 0.032
	BiLSTM	0.871 ± 0.044	0.127 ± 0.031
	TCN	0.870 ± 0.051	0.127 ± 0.036
	Friedman test effect size	0.497	0.546

**Table 11:** Pairwise comparison between Topo2DCNN-LSTM and 2DCNN-LSTM at 1.2 and 1.8 m/s walking speeds after int8 quantization.

Walking Condition	Metric	Median Difference [95%CI]	Absolute Effect Size
		Topo2DCNN-LSTM vs. 2DCNN-LSTM	
1.2 m/s	*R* ^2^	0.028 [0.016, 0.048]	0.885
	RMSE	−0.009 [−0.013, −0.006]	0.885
1.8 m/s	*R* ^2^	0.018 [0.011, 0.028]	0.885
	RMSE	−0.008 [−0.014, −0.005]	0.885

**Table 12:** RAM, flash memory and latency of the deployed model provided by Edge Impulse using EON™ Compiler based on an estimate for the Cortex-M4F 120 MHz.

	Deployment Simulation
RAM	64.7 kb
Flash	171.0 kb
Latency	19 ms

**Table 13:** On-device estimation of RAM, flash memory and latency of the deployed model provided through Arduino IDE.

Model	Available Usage	On-Device Deployment Estimation
RAM	123,143 bytes	71,316 bytes (27.2% of total usage)
Flash	507,904 bytes	257,172 bytes (50.6% of total usage)
Latency	-	28 ms

## Data Availability

The dataset used in this study is publicly available from the original repository described in [63].

## Abbreviations

The following abbreviations are used in this manuscript:

2D	Two-dimensional
2DCNN-LSTM	2D convolutional neural network–long short-term memory
3D	Three-dimensional
BF	Biceps femoris
BiLSTM	Bidirectional long short-term memory
BN	Batch Normalization
BYOM	Bring your own model
CNN	Convolutional neural network
Conv2D	2D convolution layer
EMG	Electromyography
GRU	Gated recurrent units
GRAC	Gracilis
IMU	Inertial measurement unit
int8	8-bit integer
LBF	Left biceps femoris
LGRAC	Left gracilis
LMGAS	Left medial gastrocnemius
LSTM	Long short-term memory
LVL	Left vastus lateralis
MAV	Mean absolute value
MCU	Microcontroller unit
MGAS	Medial gastrocnemius
MSE	Mean squared error
RAM	Random access memory
ReLU	Rectified linear unit
RF	Rectus femoris
RMS	Root mean square
RMSE	Root mean square error
*R*^2^	Coefficient of determination
SD	Standard deviation
TA	Tibialis anterior
TCN	Temporal convolutional network
TinyML	Tiny machine learning
VL	Vastus lateralis

## Appendix A. Statistical Analysis Summary

**Table A1:** Results of Mauchly’s Test of Sphericity with degrees of freedom of 14 and Friedman test with degrees of freedom of 5 for all models before quantization.

Walking Speeds	Metric	Mauchly’s W	Mauchly *p*-Value	Friedman χ^2^	Kendall’s W (Effect Size)	Friedman *p*-Value
1.2 m/s	RMSE	0.014	0.005	34.844	0.634	0.00000162
	*R* ^2^	0.015	0.006	37.753	0.686	0.000000423
1.8 m/s	RMSE	0.013	0.004	27.727	0.504	0.0000412
	*R* ^2^	0.006	0.001	27.727	0.504	0.0000412

**Table A2:** Results of the Holm-corrected Wilcoxon signed-rank post hoc tests for RMSE, together with Shapiro–Wilk normality tests of the paired differences, for all models before quantization.

Walking Speeds	Comparator	Median Difference (Topo2DCNN LSTM − Comparator)	[95% CI]	Absolute Effect Size	Holm-Adjusted *p*-Value	Shapiro W (*p*-Value)
1.2 m/s	LSTM	−0.018	[−0.025, −0.010]	0.885	0.005	0.898 (0.173)
	BiLSTM	−0.023	[−0.029, −0.020]	0.885	0.005	0.911 (0.252)
	2DCNN LSTM	−0.009	[−0.014, −0.006]	0.885	0.005	0.857 (0.053)
	2DCNN GRU	−0.013	[−0.018, −0.010]	0.885	0.005	0.924 (0.357)
	TCN	−0.012	[−0.018, −0.008]	0.885	0.005	0.930 (0.406)
1.8 m/s	LSTM	−0.016	[−0.021, −0.011]	0.885	0.005	0.980 (0.964)
	BiLSTM	−0.016	[−0.022, −0.010]	0.885	0.005	0.921 (0.325)
	2DCNN LSTM	−0.009	[−0.015, −0.006]	0.885	0.005	0.846 (0.037)
	2DCNN GRU	−0.012	[−0.032, −0.007]	0.885	0.005	0.788 (0.007)
	TCN	−0.020	[−0.027, −0.012]	0.858	0.005	0.971 (0.895)

**Table A3:** Results of the Holm-corrected Wilcoxon signed-rank post hoc tests for *R*^2^, together with Shapiro–Wilk normality tests of the paired differences, for all models before quantization.

Walking Speeds	Comparator	Median Difference (Topo2DCNN LSTM − Comparator)	[95% CI]	Absolute Effect Size	Holm-Adjusted *p*-Value	Shapiro W (*p*-Value)
1.2 m/s	LSTM	0.059	[0.044, 0.096]	0.885	0.005	0.817 (0.016)
	BiLSTM	0.086	[0.067, 0.109]	0.885	0.005	0.949 (0.627)
	2DCNN LSTM	0.029	[0.015, 0.047]	0.885	0.005	0.889 (0.133)
	2DCNN GRU	0.043	[0.030, 0.057]	0.885	0.005	0.852 (0.045)
	TCN	0.042	[0.031, 0.052]	0.885	0.005	0.942 (0.543)
1.8 m/s	LSTM	0.032	[0.026, 0.037]	0.885	0.005	0.959 (0.759)
	BiLSTM	0.029	[0.014, 0.044]	0.885	0.005	0.947 (0.609)
	2DCNN LSTM	0.019	[0.011, 0.031]	0.885	0.005	0.928 (0.389)
	2DCNN GRU	0.025	[0.012, 0.060]	0.885	0.005	0.722 (0.001)
	TCN	0.028	[0.019, 0.057]	0.885	0.005	0.788 (0.006)

**Table A4:** Results of Mauchly’s Test of Sphericity with degrees of freedom of 14 and Friedman test with degrees of freedom of 5 for all models after quantization.

Walking Speeds	Metric	Mauchly’s W	Mauchly *p*-Value	Friedman χ^2^	Kendall’s W (Effect Size)	Friedman *p*-Value
1.2 m/s	RMSE	0.019	0.011	37.649	0.685	0.000000444
	*R* ^2^	0.017	0.008	37.233	0.677	0.000000538
1.8 m/s	RMSE	0.013	0.004	30.013	0.546	0.0000147
	*R* ^2^	0.003	<0.0001	27.312	0.497	0.0000496

**Table A5:** Results of the Holm-corrected Wilcoxon signed-rank post hoc tests for RMSE, together with Shapiro–Wilk normality tests of the paired differences, for all models after quantization.

Walking Speeds	Comparator	Median Difference (Topo2DCNN LSTM − Comparator)	[95% CI]	Absolute Effect Size	Holm-Adjusted *p*-Value	Shapiro W (*p*-Value)
1.2 m/s	LSTM	−0.018	[−0.025, −0.011]	0.885	0.005	0.915 (0.278)
	BiLSTM	−0.024	[−0.029, −0.020]	0.885	0.005	0.945 (0.583)
	2DCNN LSTM	−0.009	[−0.013, −0.006]	0.885	0.005	0.943 (0.558)
	2DCNN GRU	−0.013	[−0.016, −0.009]	0.885	0.005	0.934 (0.453)
	TCN	−0.011	[−0.016, −0.007]	0.885	0.005	0.898 (0.176)
1.8 m/s	LSTM	−0.016	[−0.020, −0.011]	0.885	0.005	0.929 (0.406)
	BiLSTM	−0.016	[−0.022, −0.010]	0.885	0.005	0.950 (0.647)
	2DCNN LSTM	−0.008	[−0.014, −0.005]	0.885	0.005	0.822 (0.018)
	2DCNN GRU	−0.012	[−0.031, −0.006]	0.885	0.005	0.796 (0.008)
	TCN	−0.016	[−0.025, −0.010]	0.885	0.005	0.927 (0.382)

**Table A6:** Results of the Holm-corrected Wilcoxon signed-rank post hoc tests for *R*^2^, together with Shapiro–Wilk normality tests of the paired differences, across all models after quantization.

Walking Speeds	Comparator	Median Difference (Topo2DCNN LSTM − Comparator)	[95% CI]	Absolute Effect Size	Holm-Adjusted *p*-Value	Shapiro W (*p*-Value)
1.2 m/s	LSTM	0.061	[0.044, 0.093]	0.885	0.005	0.869 (0.075)
	BiLSTM	0.087	[0.069, 0.110]	0.885	0.005	0.965 (0.834)
	2DCNN LSTM	0.028	[0.016, 0.048]	0.885	0.005	0.857 (0.052)
	2DCNN GRU	0.043	[0.028, 0.054]	0.885	0.005	0.849 (0.042)
	TCN	0.039	[0.028, 0.048]	0.885	0.005	0.917 (0.293)
1.8 m/s	LSTM	0.033	[0.026, 0.037]	0.885	0.005	0.916 (0.285)
	BiLSTM	0.029	[0.015, 0.044]	0.885	0.005	0.952 (0.664)
	2DCNN LSTM	0.018	[0.011, 0.028]	0.885	0.005	0.947 (0.607)
	2DCNN GRU	0.024	[0.009, 0.058]	0.885	0.005	0.718 (0.001)
	TCN	0.023	[0.016, 0.052]	0.885	0.005	0.743 (0.002)
